# Surface Plasmon-Enhanced Photomagnetic Excitation of Spin Dynamics in Au/YIG:Co Magneto-Plasmonic Crystals

**DOI:** 10.1021/acsphotonics.1c00476

**Published:** 2021-08-06

**Authors:** Artsiom Kazlou, Alexander L. Chekhov, Alexander I. Stognij, Ilya Razdolski, Andrzej Stupakiewicz

**Affiliations:** † Faculty of Physics, University of Bialystok, 15-245 Bialystok, Poland; ‡ Department of Physics, Freie Universität Berlin, 14195 Berlin, Germany; § Scientific-Practical Materials Research Centre of the NASB, 220072 Minsk, Belarus

**Keywords:** surface plasmon-polariton, magnetization dynamics, rare-earth iron garnet, magneto-plasmonics, photomagnetic effect, nonlinear optics

## Abstract

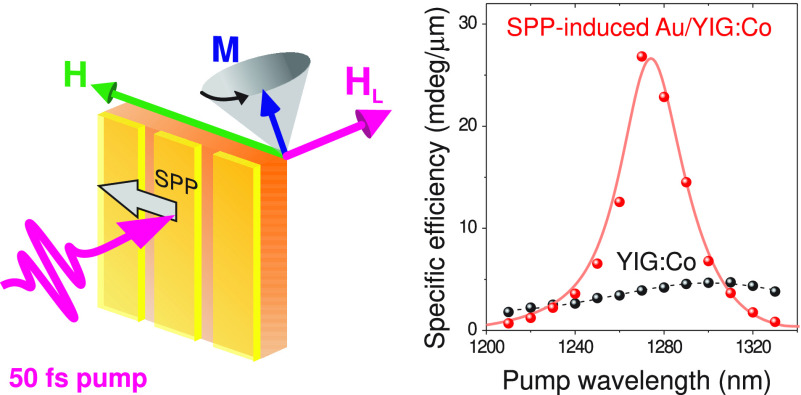

We report strong amplification of the photomagnetic spin precession in Co-doped YIG employing a surface plasmon excitation in a metal-dielectric magneto-plasmonic crystal. Plasmonic enhancement is accompanied by the localization of the excitation within the 300 nm thick layer inside the transparent dielectric garnet. Experimental results are nicely reproduced by numerical simulations of the photomagnetic excitation. Our findings demonstrate the magneto-plasmonic concept of subwavelength localization and amplification of the photomagnetic excitation in dielectric YIG:Co, which can potentially be employed for all-optical magnetization switching below the diffraction limit, with energy efficiency approaching the fundamental limit for magnetic memories.

In the past decade, the rapid progress of ultrafast optomagnetism has opened up rich possibilities for optical data recording in magnetic materials, aiming at the heat-assisted magnetic recording with 20 × 20 × 10 nm bit size in metallic media.^1^ Meanwhile, the research has been fueled into alternative approaches, including the highly promising all-optical magnetization switching demonstrated in multiple metallic systems in the past few years.^2,3^ However, the all-optical switching in metals requires heating to high temperatures and demagnetization.^4^ Recently, based on a direct modification of the magnetic anisotropic energy barrier,^5−7^ a nonthermal method for ultrafast photomagnetic recording in dielectric garnets has been developed^8^ employing a magnetization precession mechanism.^9,10^ There, the key to future applications is the optics of photomagnetic recording with the light localization into a ∼ 20 nm size spot, thus, approaching the Landauer limit (∼0.25 aJ).^11^

This challenge of confining the photoexcitation within subwavelength volumes can be addressed with magneto-plasmonics, a rapidly developing branch of modern photonics.^12−14^ The high potential of magneto-plasmonics for local manipulation of magnetic order with photons is already established.^15,16^ On the other hand, a highly interesting class of systems emerged recently, where magnetic dielectrics are covered with gratings made of plasmonic metals (such as Au).^17−19^ The grating allows for the free-space excitation of surface plasmon polaritons (SPPs) at both interfaces of the metal,^20,21^ whereas the transparent dielectric layer ensures low losses of the SPP excitations, in contrast to magneto-plasmonic systems with transition metal ferromagnets.^22^ Tailoring the electric field distribution inside the dielectric through the metal-bound SPP excitation enables novel nonlinear-optical and opto-magnetic effects.^23−35^

In this work, we employ this magneto-plasmonic grating approach for amplifying the photomagnetic spin precession in a dielectric Co-doped yittrium iron garnet (YIG:Co). We observe a strong increase of the magnetization precession amplitude in the vicinity of the SPP resonance in the near-infrared. Numerical simulations of the electric field distribution show that, due to the SPP-induced light localization at the interface, the specific efficiency of the excitation of the magnetization precession is enhanced 6-fold within the 300 nm active layer, as compared to the bare garnet film. Because photomagnetic switching is a threshold effect, our results represent an important step toward nanoscale photomagnetic data writing with femtosecond laser pulses. They highlight the rich potential of the magneto-plasmonic approach for scaling down toward nm-sized magnetic bits and further improving the energy efficiency of the all-optical magnetic recording.

## Experimental Details

Experimental studies of the SPP-induced photomagnetic anisotropy were performed on Au/YIG:Co magneto-plasmonic crystals consisting of a 7.5 μm thick garnet film covered with Au gratings.^36^ YIG:Co is a weakly opaque (in the near-infrared spectral range) ferrimagnet with a saturation magnetization of 4π*M*_s_ = 80 Gs and a Neel temperature of 455 K. The Co dopants display strong single ion anisotropy, which depends on the ion’s valence state. Therefore, resonant pumping of Co electronic transitions with laser pulses enables direct access to the magnetic anisotropy and, thus, the magnetization. This results in the exceptionally strong photomagnetic effect in YIG:Co,^7^ ultimately allowing ultrafast magnetization switching with a single femtosecond laser pulse.^8^ The Co doping also enhances magnetocrystalline anisotropy and the Gilbert damping α = 0.2.^8^ The YIG:Co garnet film with a composition of Y_2_CaFe_3.9_Co_0.1_GeO_12_ was grown on a Gd_3_Ga_5_O_12_ (001) substrate. The surface of the garnet thin film was treated with a low-energy oxygen ion beam.^37^ A 50 nm thick Au grating with an 800 nm period (gap width 100 nm) was deposited on the garnet surface by ion-beam sputtering and perforated using FIB.^38^

**Figure 1:**
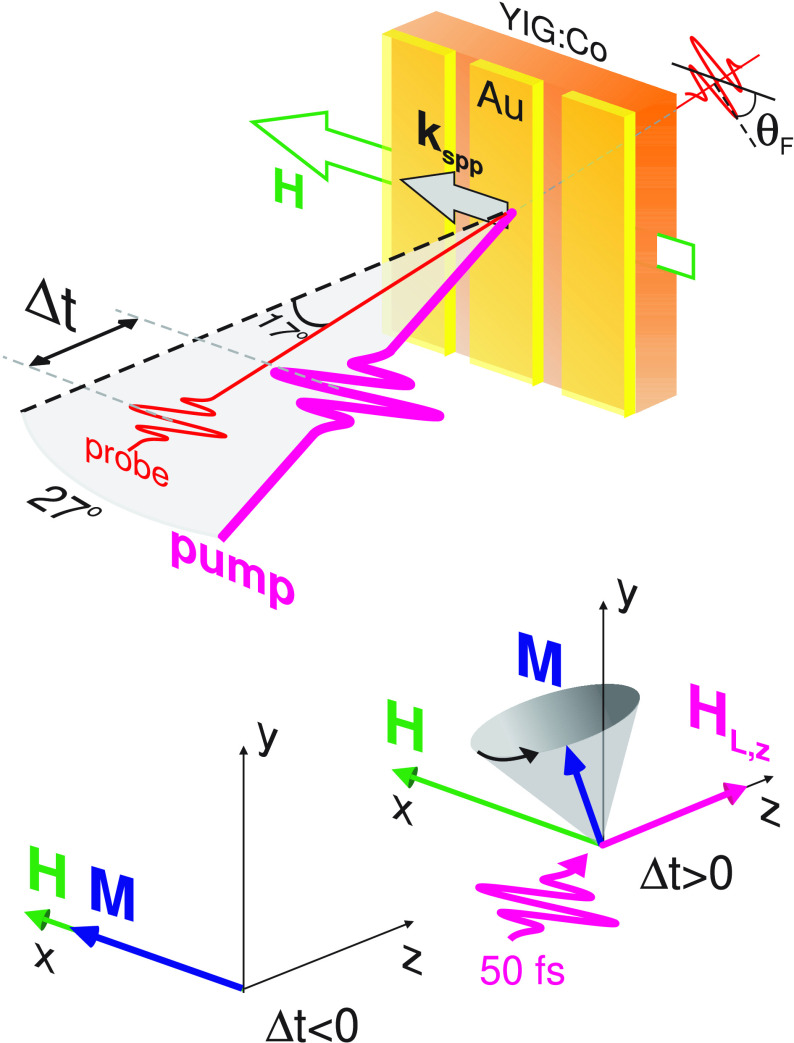
Schematics of the experimental pump–probe transmission geometry. The 50 fs long near-IR *p*-polarized pump pulses excite a SPP resonance at the Au/YIG:Co interface. The electromagnetic SPP field induces the photomagnetic anisotropy **H**_**L**_ in YIG:Co triggering the magnetization precession. The latter is monitored through transient Faraday rotation θ_F_ of the delayed probe pulses.

SPP-driven photomagnetic excitation of magnetization precession was studied in the two-color pump–probe transmission geometry schematically shown in Figure 1. There, the time-resolved Faraday rotation angle θ_F_ of the probe beam was monitored as a function of the delay time Δ*t* between the pump and probe pulses. The pump (probe) laser pulses with a duration of 50 fs from a Ti-Sapphire amplifier at a 500 Hz (1 kHz) repetition rate impinged at an angle of 27° (17°) from the sample normal, respectively, see Figure 1. Employing the optical parametric amplifier, the wavelength of the pump beam λ was tuned in the near-infrared range within 1200–1350 nm, while the probe wavelength was set to 800 nm. The more powerful pump beam was focused into a spot of about 100 μm in diameter on the samples, resulting in an energy density of ∼4 mJ/cm^2^, while the spot size of the 30 times weaker probe beam was half the size. Both pump and probe beams were polarized in their plane of incidence. The delay time Δ*t* between the pump and probe pulses was controlled by means of a motorized delay stage.

An external magnetic field *H* = 3.2 kOe was applied in-plane of the sample along the [100] direction of the garnet crystal (Figure 1) to set the magnetization **M**∥**H**. This enables monitoring the magnetization precession through the transient Faraday rotation θ_F_ proportional to the out-of-plane component *M*_*z*_. It has been shown earlier that, in this Co-doped garnet, the photomagnetic excitation is the dominant effect in the near-infrared spectral range.^7−9^ This is in contrast with Bi-, Lu-, and other rare-earth-doped garnets, demonstrating an opto-magnetic inverse Faraday effect. In our geometry, a torque  is exerted on **M** by the effective field of the photoinduced magnetic anisotropy in YIG:Co **H**_**L**_ = χ̂⋮**EEM**, where **E** is the electric field of light and χ̂ is the photomagnetic third order susceptibility tensor.^8^ This torque  triggers the magnetization precession around its new equilibrium determined by **H**, **H**_**L**_, and the magneto-crystalline anisotropy field **H**_**c**_. At laser fluences used in this work, we estimate *H*_*L*_ ∼ 10^2^ Oe, resulting in a tilt of the equilibrium a few degrees away from the in-plane direction of **H**. After the relaxation of **H**_**L**_ (on the scale of 20 ps^7,8^), the equilibrium position for the magnetization is restored to its initial direction ∥**H**, and its precession proceeds around the original effective field direction. Because the incident light pulse is *p*-polarized with the electric field **E** = (*E*_*x*_, 0, *E*_*z*_) and the garnet has the cubic 4*mm* symmetry, a nonzero torque on *M*_*x*_ is generated by the following component of **H**_**L**_:
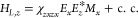
1Notably, because the refractive index of garnets is relatively large (*n* ≳ 2 in the near-infrared^39^), the surface normal projection of the electric field *E*_*z*_ of the propagating light is suppressed. On the contrary, being one of the characteristic features of the SPP excitation at a metal–dielectric interface, prominent enhancement of *E*_*z*_ in the dielectric promises an amplification of the photomagnetic anisotropy field and, thus, large angles of magnetization precession.

We studied the magnetization precession in the spectral vicinity of the SPP resonance at the Au/YIG:Co interface (∼1275 nm at 27° of incidence^29,36^). At each pump wavelength λ, we measured the transient rotation of the probe polarization θ at the two opposite directions of **H** and analyzed their difference, thus, removing concomitant signal variations of a nonmagnetic origin. To verify the symmetry and the magnitude of the photomagnetic effect and enable a reference point, similar measurements were performed on a bare YIG:Co film without an Au grating.

## Experimental Results

**Figure 2:**
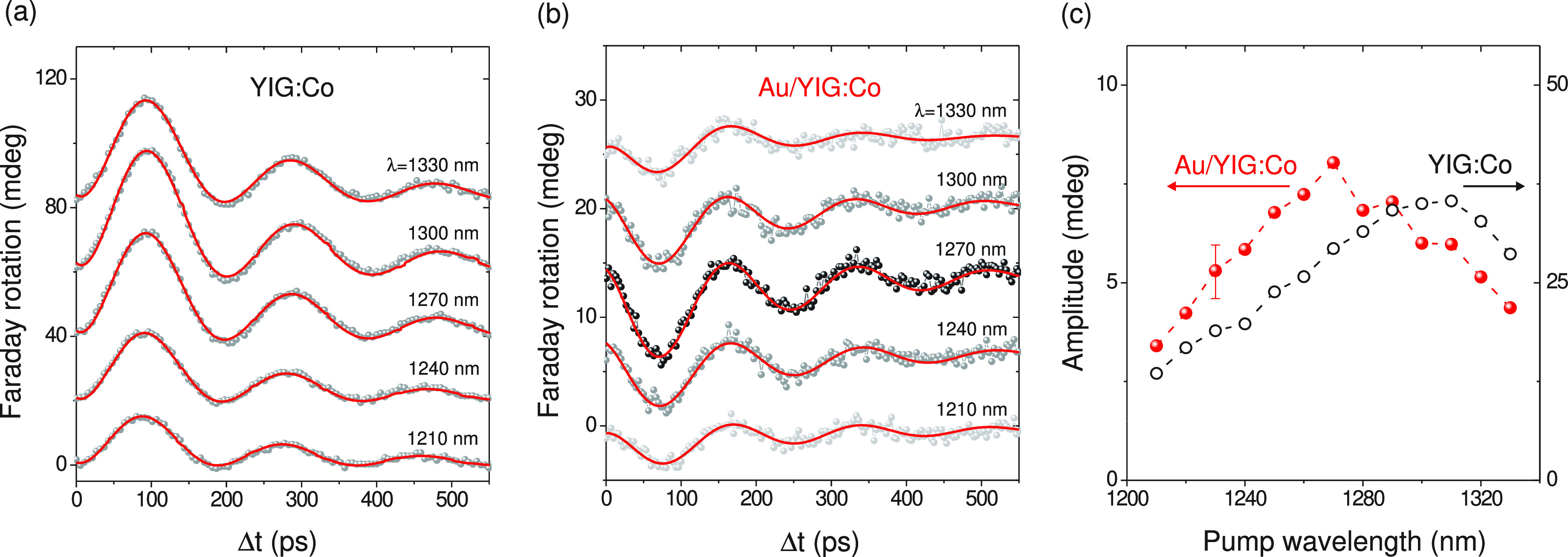
Time-resolved Faraday rotation in bare YIG:Co film (a) and Au/YIG:Co magneto-plasmonic crystal (b) induced by pump pulses of varied wavelength. The data sets are shifted vertically without rescaling. The red lines show the single-frequency damped sine function fitted to the data. (c) Spectral dependence of the Faraday rotation amplitude extracted from the fits shown in panels (a) and (b).

The time-resolved Faraday rotation traces θ_F_ = [θ(+*H*) – θ(−*H*)]/2 obtained on the bare garnet and the Au/YIG:Co magneto-plasmonic crystal are shown in Figure 2a and b, respectively. It is seen that both samples demonstrate similar magnetization dynamics which can be reasonably well described by a single-mode precession at about 5 GHz frequency, in agreement with the previous findings.^7^ Other temporal details of the magnetization dynamics will be discussed in a subsequent publication, and here we only focus on the precession amplitude. It can be further seen in Figure 2 that the absolute amplitude in the YIG:Co sample is approximately 1 order of magnitude stronger than that in the magneto-plasmonic Au:YIG:Co crystal. This reduction of the precession magnitude originates in the smaller pump energy reaching the garnet layer (due to significant reflectivity of the grating enabled by the <100% coupling efficiency of the free-space incident radiation and the SPP) and strong SPP-driven localization of the excitation in the perpendicular direction. Owing to the latter, only a thin layer adjacent to the Au/YIG:Co interface significantly contributes to the transient Faraday rotation, in contrast with the bare garnet where θ_F_ is accumulated across its entire thickness (7.5 um). This indicates the importance of considering an effective depth contributing to the transient Faraday rotation, which will be introduced below. It is already seen that the spectral behavior of these two samples differs noticeably (Figure 2c). Indeed, in the bare garnet the precession is slightly enhanced at around λ ≈ 1300 nm, whereas the Au/YIG:Co exhibits a different spectral shape, with the largest precession amplitude observed around λ ≈ 1270 nm (highlighted with darker points in Figure 2b).

## Numerical Simulations

**Figure 3:**
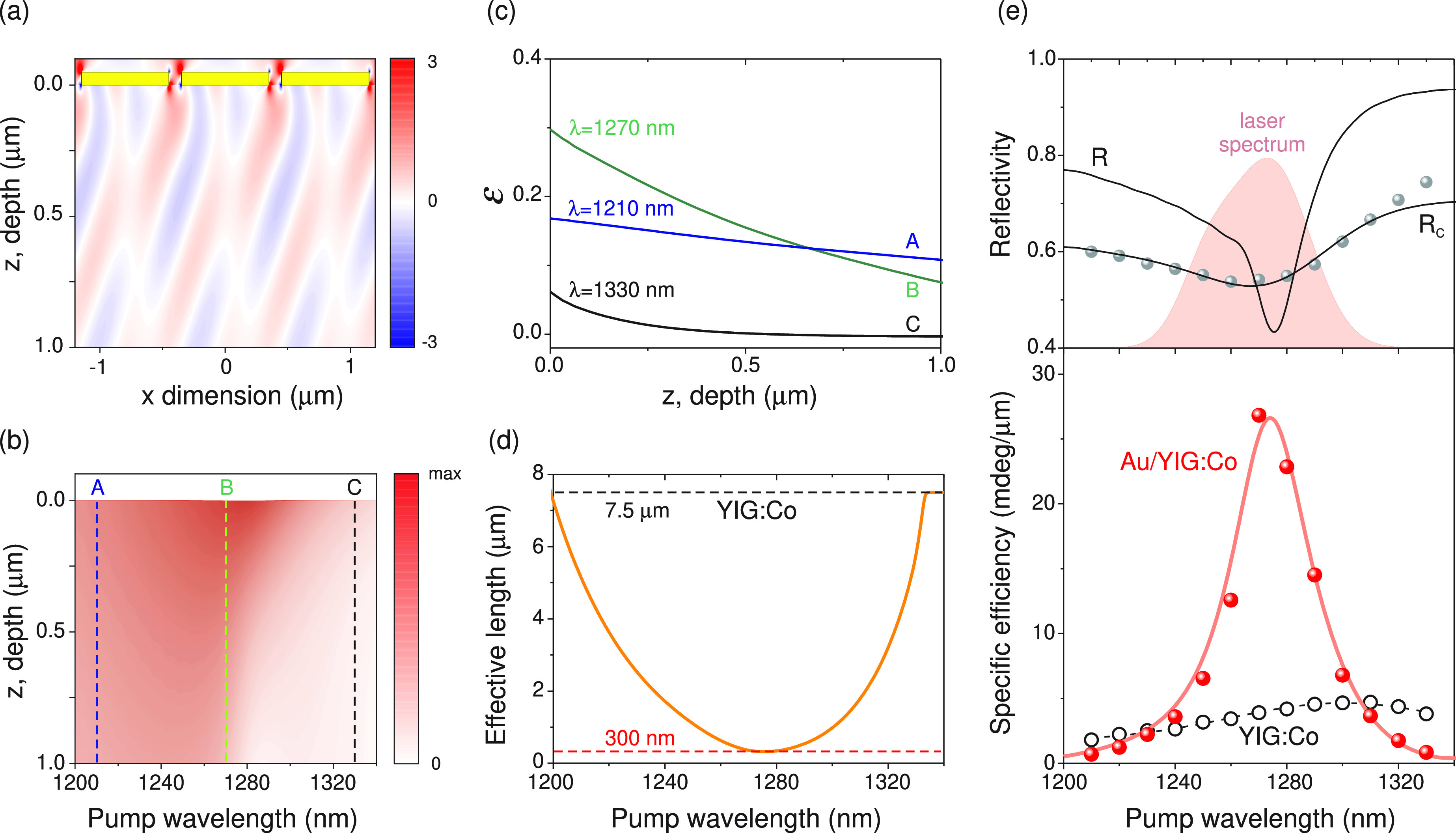
(a) Numerically simulated spatial distribution (*x*,*z*) inside the YIG:Co layer at the SPP resonance (λ = 1270 nm). The yellow bars indicate the Au grating. (b, c) Depth profiles (*z*,λ) integrated across the *x*-dimension. The dashed lines indicate three λ values that are shown in (c) in detail; *B* (λ = 1270 nm) corresponds to the resonant SPP excitation. (d) Calculated effective decay depth *l*_eff_ of the photomagnetic excitation away from the Au/YIG:Co interface. The top dashed line at 7.5 μm indicates the total thickness of the YIG:Co layer. The bottom dashed line at 300 nm shows the shortest effective depth obtained at the resonance. (e) (top) Calculated reflectivity spectra, before (*R*) and after (*R*_c_) taking into account the spectral broadening due to the finite laser pulse duration. Full dots: experimental Au/YIG:Co reflectivity. The shaded area illustrates the measured laser spectrum at around λ = 1270 nm. (bottom) Specific efficiency of the photomagnetic excitation for the bare garnet (open dots) and Au:YIG/Co (full dots). The solid red line is the result of the numerical simulations.

To quantify the SPP-induced enhancement of the precession amplitude, we performed numerical simulations of the SPP-driven excitation employing dedicated Lumerical software.^40^ From eq 1 it can be shown that the photoinduced anisotropy field *H*_*L*_ takes the following form:
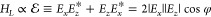
2where  shows the excitation magnitude, φ is the phase shift between the two components of the electric field *E*_*x*_, *E*_*z*_, and the asterisk indicates the complex conjugate. We calculated  inside the YIG:Co layer in the vicinity of the SPP resonance. Optical constants of Au were taken from ref (41). Figure 3 shows the characteristic spatial distribution of (*x*,*z*,λ) calculated for the λ = 1270 nm, that is, at the SPP resonance. In the figure, we only show the top 1 μm thick layer of YIG:Co, although the calculations were performed for the entire garnet film. These data maps were collected for a set of λ and, for each of them, integrated across the *x*-axes to enable the spectral comparison of the depth distributions (*z*,λ). Those results are shown in Figure 3b, where the darker shaded regions illustrate the enhancement and interfacial localization of the optical excitation. To highlight the latter point, we show a few selected depth profiles (indicated with dashed lines in Figure 3b) in Figure 3c. At each λ, from these profiles we calculated the effective excitation depth *l*_eff_ = |·(d/d*z*)^−1^|_*z*=0_ containing the most significant part of the excitation energy (see in Figure 3d). It is seen there that, at the SPP resonance, the excitation is concentrated within the 300 nm layer adjacent to the Au–garnet interface. On the contrary, away from the resonance, the effective depth *l*_eff_ increases rapidly toward the total thickness of the YIG:Co layer (7.5 μm).

Finally, in order to compare the results of our calculations with the experimental data, spectral broadening due to the finite laser pulse duration has to be taken into account. Indeed, with 50 fs-short laser pulses, every experimental wavelength shown in Figure 2 in fact represents a continuum of wavelengths around the indicated value. A characteristic 50 nm wide spectrum of a laser pulse *S*(λ) centered around λ = 1270 nm is exemplified in the top panel of Figure 3e with the shaded area. To verify the broadening, we compared the experimental reflectivity spectrum of the Au/YIG:Co magneto-plasmonic crystal and the calculated data *R*(λ) convoluted with *S*(λ): . A very good agreement with the experimental data allows us to apply the same procedure to the calculated depth-integrated (λ) = ∫(*z*, λ)d*z* data to obtain specific excitation efficiency ξ(λ) = [(λ)χ(λ)]*S(λ)/*l*_eff_(λ). Here, χ(λ) accounts for the dispersion of the photomagnetic tensor χ̂ discussed earlier, which can be extracted from the spectral dependence of the precession amplitude obtained on a bare YIG:Co.

The results of this procedure are summarized in the bottom panel of Figure 3e. There, we also show the specific excitation efficiency for the bare transparent garnet, assuming that the effective depth equals its total thickness of 7.5 μm. Strong enhancement of the efficiency at the SPP resonance in the Au/YIG:Co sample is in a striking contrast with the flat spectral dependence on a bare garnet. It is seen that the SPP excitation results in the 6-fold enhancement of the specific amplitude of the magnetization precession at the resonance.

## Discussion

It is worth emphasizing the similarities and differences between the systems studied here and in our recent work.^29^ In both experiments, strong amplification of the spin dynamics is inherently related to the SPP-driven localization of light at the interface. Together with the enhancement of the SPP electric fields, this effect is generic for the entire class of metal–dielectric plasmonic heterostructures and can be further optimized for better performance. From the photonic point of view, another common important impact of the SPP excitation consists in the amplification of the out-of-plane projection of the electric field *E*_*z*_, which is otherwise suppressed in the high-*n* dielectric.

The tensorial character of eq 1 reveals important differences between the two cases. In general, the following form for the effective opto-magnetic field *H*_eff_ can be derived:^42^
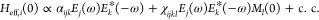
3where higher-order (in *M*) terms are neglected and α_*ijk*_ and χ_*ijkl*_ are the antisymmetric and symmetric susceptibility tensors, respectively.^43^ The symmetry determines their dependence on the phase shift φ and thus on the polarization of light. The inverse Faraday effect captured by the first term in eq 3 requires φ ≠ 0 which can be realized by employing either circularly polarized light or SPP excitation.^29,44^ On the contrary, the photomagnetic effect is present even in the absence of SPP, and moreover, the purely SPP-driven photomagnetic contribution vanishes due to φ_SPP_ = π/2 (cf. eq 2). However, due to the partial (<100%) coupling of the free-space radiation with the SPP and relatively rapid (<1 ps) SPP radiative decay enabled by the grating, the electromagnetic field *E* inside the garnet layer is formed by the interference of the incident (SPP-decoupled) and the SPP fields of comparable magnitudes. Thus, a significant phase shift φ between the two field projections appears in the vicinity of the SPP resonance, enabling the photomagnetic effect described above. To verify this, we calculated the spectra of the phases of the electric field components and the phase shift φ(λ) at the Au/YIG:Co interface. These spectra exemplified in Supporting Information indeed demonstrate a significant phase shift φ around λ = 1275 nm.

We argue that this phase shift is driven by the SPP excitation at the Au/YIG:Co interface. Corroborated by the strong localization and enhancement of the electric field at the interface, this effect is responsible for the photomagnetic SPP-mediated mechanism of the excitation, whereas its magnitude  is determined by the optical interference of the SPP and incident fields. Similarly, the reversal of the precession phase when comparing magneto-plasmonic crystals with the bare garnet, as seen in Figure 2, can also be attributed to this interference of the electromagnetic fields.

The excitation mechanism of the spin dynamics here originates in the Co doping of YIG, enabling setting magnetization into motion through an effective SPP-mediated photomagnetic anisotropy field. The photomagnetic mechanism offers the largest angles of magnetization precession available up to date at nondestructive laser fluences.^7−10^ Recalculation of the observed θ_F_ using the static Faraday rotation values measured independently (see Supporting Information for hysteresis loops) into precession angles yields about 5° magnetization excursion from the equilibrium, 1–2 orders of magnitude larger than that found in Gd,Yb-doped BIG^29^ and LuIG.^6^ It can be conjectured that further amplification is feasible through structural optimization of the plasmonic geometry aimed at enhancing the excitation  and employing numerical simulations. In particular, improvement of the SPP coupling efficiency and localization of the excitation are promising directions for further amplification of the magnetization excursion toward its reversal.

From the perspective of magnetic data recording, the photomagnetic switching through large-angle precession has been demonstrated exclusively in YIG:Co. The SPP photomagnetic mechanism thus has good potential for taking the all-optical magnetization switching onto the nanoscale. In our prototype system, the observed 6-fold amplification of the specific efficiency ξ allows for a corresponding reduction of the laser fluence below the switching threshold. The extrapolation to nm-sized bits yields about only ∼2 aJ of deposited energy per one bit, close to the fundamental thermodynamical limit for switching magnetic bits, thus, corroborating the exceptional energy efficiency of the photomagnetic nanosize switching.

## Supporting Information Available

Calculated spectra of the phases of the electric field at the Au/YIG:Co interface. Faraday rotation hysteresis loops obtained on bare YIG:Co and on Au/YIG:Co magneto-plasmonic crystal (PDF)

## References

[ref1] Challener W. A.; Peng C.; Itagi A. V.; Karns D.; Peng W.; Peng Y.; Yang X.; Zhu X.; Gokemeijer N. J.; Hsia Y.-T.; Ju G.; Rottmayer R. E.; Seigler M. A.; Gage E. C. Heat-assisted magnetic recording by a near-field transducer with efficient optical energy transfer. Nat. Photonics 2009, 3, 220–224. 10.1038/nphoton.2009.26.

[ref2] Stanciu C. D.; Hansteen F.; Kimel A. V.; Kirilyuk A.; Tsukamoto A.; Itoh A.; Rasing T. All-Optical Magnetic Recording with Circularly Polarized Light. Phys. Rev. Lett. 2007, 99, 047601 10.1103/PhysRevLett.99.047601. 17678404

[ref3] Lambert C.-H.; Mangin S.; Varaprasad B. S. D. C. S.; Takahashi Y. K.; Hehn M.; Cinchetti M.; Malinowski G.; Hono K.; Fainman Y.; Aeschlimann M.; Fullerton E. E. All-optical control of ferromagnetic thin films and nanostructures. Science 2014, 345, 1337 10.1126/science.1253493. 25147280

[ref4] Kirilyuk A.; Kimel A. V.; Rasing T. Ultrafast optical manipulation of magnetic order. Rev. Mod. Phys. 2010, 82, 2731–2784. 10.1103/RevModPhys.82.2731.

[ref5] Stupakiewicz A.; Maziewski A.; Davidenko I.; Zablotskii V. Light-induced magnetic anisotropy in Co-doped garnet films. Phys. Rev. B: Condens. Matter Mater. Phys. 2001, 64, 064405 10.1103/PhysRevB.64.064405.

[ref6] Hansteen F.; Kimel A.; Kirilyuk A.; Rasing T. Femtosecond Photomagnetic Switching of Spins in Ferrimagnetic Garnet Films. Phys. Rev. Lett. 2005, 95, 047402 10.1103/PhysRevLett.95.047402. 16090839

[ref7] Atoneche F.; Kalashnikova A. M.; Kimel A. V.; Stupakiewicz A.; Maziewski A.; Kirilyuk A.; Rasing T. Large ultrafast photoinduced magnetic anisotropy in a cobalt-substituted yttrium iron garnet. Phys. Rev. B: Condens. Matter Mater. Phys. 2010, 81, 214440 10.1103/PhysRevB.81.214440.

[ref8] Stupakiewicz A.; Szerenos K.; Afanasiev D.; Kirilyuk A.; Kimel A. V. Ultrafast nonthermal photo-magnetic recording in a transparent medium. Nature 2017, 542, 71–74. 10.1038/nature20807. 28099412 PMC5292041

[ref9] Stupakiewicz A.; Szerenos K.; Davydova M. D.; Zvezdin K. A.; Zvezdin A. K.; Kirilyuk A.; Kimel A. V. Selection rules for all-optical magnetic recording in iron garnet. Nat. Commun. 2019, 10, 612 10.1038/s41467-019-08458-w. 30723207 PMC6363756

[ref10] Szerenos K.; Kimel A.; Maziewski A.; Kirilyuk A.; Stupakiewicz A. Fundamental Limits on the Repetition Rate of Photomagnetic Recording. Phys. Rev. Appl. 2019, 12, 044057 10.1103/PhysRevApplied.12.044057.

[ref11] Landauer R. Irreversibility and Heat Generation in the Computing Process. IBM J. Res. Dev. 1961, 5, 183–191. 10.1147/rd.53.0183.

[ref12] Armelles G.; Cebollada A.; García-Martín A.; González M. U. Magnetoplasmonics: Combining Magnetic and Plasmonic Functionalities. Adv. Opt. Mater. 2013, 1, 10–35. 10.1002/adom.201200011.

[ref13] Belotelov V. I.; Kalish A. N.; Zvezdin A. K. Digital Encyclopedia of Applied Physics; American Cancer Society, 2019; pp 1–24.

[ref14] Maccaferri N.; Zubritskaya I.; Razdolski I.; Chioar I.-A.; Belotelov V.; Kapaklis V.; Oppeneer P. M.; Dmitriev A. Nanoscale magnetophotonics. J. Appl. Phys. 2020, 127, 080903 10.1063/1.5100826.

[ref15] Liu T.-M.; et al. Nanoscale Confinement of All-Optical Magnetic Switching in TbFeCo - Competition with Nanoscale Heterogeneity. Nano Lett. 2015, 15, 6862–6868. 10.1021/acs.nanolett.5b02743. 26312732

[ref16] von Korff Schmising C.; Giovannella M.; Weder D.; Schaffert S.; Webb J. L.; Eisebitt S. Nonlocal ultrafast demagnetization dynamics of Co/Pt multilayers by optical field enhancement. New J. Phys. 2015, 17, 033047 10.1088/1367-2630/17/3/033047.

[ref17] Sepulveda B.; Lechuga L. M.; Armelles G. Magnetooptic effects in surface-plasmon-polaritons slab waveguides. J. Lightwave Technol. 2006, 24, 945–955. 10.1109/JLT.2005.861943.

[ref18] Wurtz G. A.; Hendren W.; Pollard R.; Atkinson R.; Guyader L. L.; Kirilyuk A.; Rasing T.; Smolyaninov I. I.; Zayats A. V. Controlling optical transmission through magneto-plasmonic crystals with an external magnetic field. New J. Phys. 2008, 10, 105012 10.1088/1367-2630/10/10/105012.

[ref19] Belotelov V. I.; Akimov I. A.; Pohl M.; Kotov V. A.; Kasture S.; Vengurlekar A. S.; Gopal A. V.; Yakovlev D. R.; Zvezdin A. K.; Bayer M. Enhanced magneto-optical effects in magnetoplasmonic crystals. Nat. Nanotechnol. 2011, 6, 370–376. 10.1038/nnano.2011.54. 21516090

[ref20] Pohl M.; Kreilkamp L. E.; Belotelov V. I.; Akimov I. A.; Kalish A. N.; Khokhlov N. E.; Yallapragada V. J.; Gopal A. V.; Nur-E-Alam M.; Vasiliev M.; Yakovlev D. R.; Alameh K.; Zvezdin A. K.; Bayer M. Tuning of the transverse magneto-optical Kerr effect in magneto-plasmonic crystals. New J. Phys. 2013, 15, 075024 10.1088/1367-2630/15/7/075024.

[ref21] Razdolski I.; Parchenko S.; Stupakiewicz A.; Semin S.; Stognij A.; Maziewski A.; Kirilyuk A.; Rasing T. Second-Harmonic Generation from a Magnetic Buried Interface Enhanced by an Interplay of Surface Plasma Resonances. ACS Photonics 2015, 2, 20–26. 10.1021/ph500382u.

[ref22] Temnov V. V.; Armelles G.; Woggon U.; Guzatov D.; Cebollada A.; Garcia-Martin A.; Garcia-Martin J.-M.; Thomay T.; Leitenstorfer A.; Bratschitsch R. Active magneto-plasmonics in hybrid metal–ferromagnet structures. Nat. Photonics 2010, 4, 107–111. 10.1038/nphoton.2009.265.

[ref23] Khurgin J. B. Optical isolating action in surface plasmon polaritons. Appl. Phys. Lett. 2006, 89, 251115 10.1063/1.2422885.

[ref24] Krutyanskiy V. L.; Chekhov A. L.; Ketsko V. A.; Stognij A. I.; Murzina T. V. Giant nonlinear magneto-optical response of magnetoplasmonic crystals. Phys. Rev. B: Condens. Matter Mater. Phys. 2015, 91, 121411 10.1103/PhysRevB.91.121411.

[ref25] Chekhov A. L.; Razdolski I.; Kirilyuk A.; Rasing T.; Stognij A. I.; Murzina T. V. Surface plasmon-driven second-harmonic generation asymmetry in anisotropic plasmonic crystals. Phys. Rev. B: Condens. Matter Mater. Phys. 2016, 93, 161405 10.1103/PhysRevB.93.161405.

[ref26] Girón-Sedas J. A.; Reyes Gómez F.; Albella P.; Mejía-Salazar J. R.; Oliveira O. N. Giant enhancement of the transverse magneto-optical Kerr effect through the coupling of ε-near-zero and surface plasmon polariton modes. Phys. Rev. B: Condens. Matter Mater. Phys. 2017, 96, 075415 10.1103/PhysRevB.96.075415.

[ref27] Im S.-J.; Ri C.-S.; Ho K.-S.; Herrmann J. Third-order nonlinearity by the inverse Faraday effect in planar magnetoplasmonic structures. Phys. Rev. B: Condens. Matter Mater. Phys. 2017, 96, 165437 10.1103/PhysRevB.96.165437.

[ref28] Ho K.-S.; Im S.-J.; Pae J.-S.; Ri C.-S.; Han Y.-H.; Herrmann J. Switchable plasmonic routers controlled by external magnetic fields by using magneto-plasmonic waveguides. Sci. Rep. 2018, 8, 10584 10.1038/s41598-018-28567-8. 30002560 PMC6043596

[ref29] Chekhov A. L.; Stognij A. I.; Satoh T.; Murzina T. V.; Razdolski I.; Stupakiewicz A. Surface Plasmon-Mediated Nanoscale Localization of Laser-Driven sub-Terahertz Spin Dynamics in Magnetic Dielectrics. Nano Lett. 2018, 18, 2970–2975. 10.1021/acs.nanolett.8b00416. 29641902

[ref30] Im S.-J.; Pae J.-S.; Ri C.-S.; Ho K.-S.; Herrmann J. All-optical magnetization switching by counterpropagataion or two-frequency pulses using the plasmon-induced inverse Faraday effect in magnetoplasmonic structures. Phys. Rev. B: Condens. Matter Mater. Phys. 2019, 99, 041401 10.1103/PhysRevB.99.041401.

[ref31] Barsukova M. G.; Musorin A. I.; Shorokhov A. S.; Fedyanin A. A. Enhanced magneto-optical effects in hybrid Ni-Si metasurfaces. APL Photonics 2019, 4, 016102 10.1063/1.5066307.

[ref32] Pae J.-S.; Im S.-J.; Song K.-S.; Ri C.-S.; Ho K.-S.; Han Y.-H.; Herrmann J. Deep subwavelength flow-resonant modes in a waveguide-coupled plasmonic nanocavity. Phys. Rev. B: Condens. Matter Mater. Phys. 2020, 101, 245420 10.1103/PhysRevB.101.245420.

[ref33] Strelniker Y. M.; Bergman D. J. Surface versus localized plasmons in an assembly of metal-dielectric parallel flat slabs in the presence of an in-plane magnetic field. Phys. Rev. B: Condens. Matter Mater. Phys. 2020, 102, 035302 10.1103/PhysRevB.102.035302.

[ref34] López-Ortega A.; Zapata-Herrera M.; Maccaferri N.; Pancaldi M.; Garcia M.; Chuvilin A.; Vavassori P. Enhanced magnetic modulation of light polarization exploiting hybridization with multipolar dark plasmons in magnetoplasmonic nanocavities. Light: Sci. Appl. 2020, 9, 49 10.1038/s41377-020-0285-0. 32257180 PMC7105458

[ref35] Strelniker Y. M.; Bergman D. J. Itinerant versus localized plasmons in an assembly of metal-dielectric parallel flat slabs in the presence of a perpendicular magnetic field: Faraday and magneto-optical Kerr effects. Phys. Rev. B: Condens. Matter Mater. Phys. 2021, 103, 205302 10.1103/PhysRevB.103.205302.

[ref36] Razdolski I.; Chekhov A. L.; Stognij A. I.; Stupakiewicz A. Ultrafast transport and relaxation of hot plasmonic electrons in metal-dielectric heterostructures. Phys. Rev. B: Condens. Matter Mater. Phys. 2019, 100, 045412 10.1103/PhysRevB.100.045412.

[ref37] Pashkevich M.; Gieniusz R.; Stognij A.; Novitskii N.; Maziewski A.; Stupakiewicz A. Formation of cobalt/garnet heterostructures and their magnetic properties. Thin Solid Films 2014, 556, 464–469. 10.1016/j.tsf.2014.01.064.

[ref38] Chekhov A. L.; Krutyanskiy V. L.; Ketsko V. A.; Stognij A. I.; Murzina T. V. High-quality Au/BIG/GGG magnetoplasmonic crystals fabricated by a combined ion-beam etching technique. Opt. Mater. Express 2015, 5, 1647–1652. 10.1364/OME.5.001647.

[ref39] Landolt H.; Börnstein R. Numerical Data and Functional Relationships in Science and Technology; New series, Group III, 27th edition; Springer: Berlin, 1991; pp 75–76.

[ref40] Lumerical Solutions, Inc.: Vancouver, BC V6E 3L2, Canada, https://www.lumerical.com/.

[ref41] Johnson P. B.; Christy R. W. Optical Constants of the Noble Metals. Phys. Rev. B 1972, 6, 4370–4379. 10.1103/PhysRevB.6.4370.

[ref42] Kimel A. V.; Zvezdin A. K. Magnetization dynamics induced by femtosecond light pulses. Low Temp. Phys. 2015, 41, 682–688. 10.1063/1.4931650.

[ref43] Landau L. D.; Lifshitz E. M. Statistical Physics. Part 1. Theoretical Physics; Nauka: Moscow, 1976; Vol. 5; pp 397–400.

[ref44] Khokhlov N.; Belotelov V.; Kalish A.; Zvezdin A. Surface Plasmon Polaritons and Inverse Faraday Effect. Solid State Phenom. 2012, 190, 369–372. 10.4028/www.scientific.net/SSP.190.369.

