# Supplementary material for: Surface Plasmon-Enhanced Photomagnetic Excitation of Spin Dynamics in Au/YIG:Co Magneto-Plasmonic Crystals
Source: ACS Photonics. 2021 Aug 6;8(8):2197–202. doi: 10.1021/acsphotonics.1c00476 (PMC8383306; doi:10.1021/acsphotonics.1c00476)
Supplement: Supplementary file 1 — ph1c00476_si_001.pdf [file ph1c00476_si_001.pdf]

Surface plasmon-enhanced photo-magnetic excitation of spin dynamics in Au/YIG:Co magneto-plasmonic crystals: Supporting Information

Artsiom Kazlou,[†] Alexander L. Chekhov,[‡] Alexander I. Stognij,[¶] Ilya Razdolski,[†] and Andrzej Stupakiewicz[*,†]

†*Faculty of Physics, University of Bialystok, 15-245 Bialystok, Poland*

‡*Department of Physics, Freie Universität Berlin, 14195 Berlin, Germany*

¶*Scientific-Practical Materials Research Centre of the NASB, 220072 Minsk, Belarus*

E-mail: and@uwb.edu.pl

This Supporting Information contains 3 pages and 2 figures.

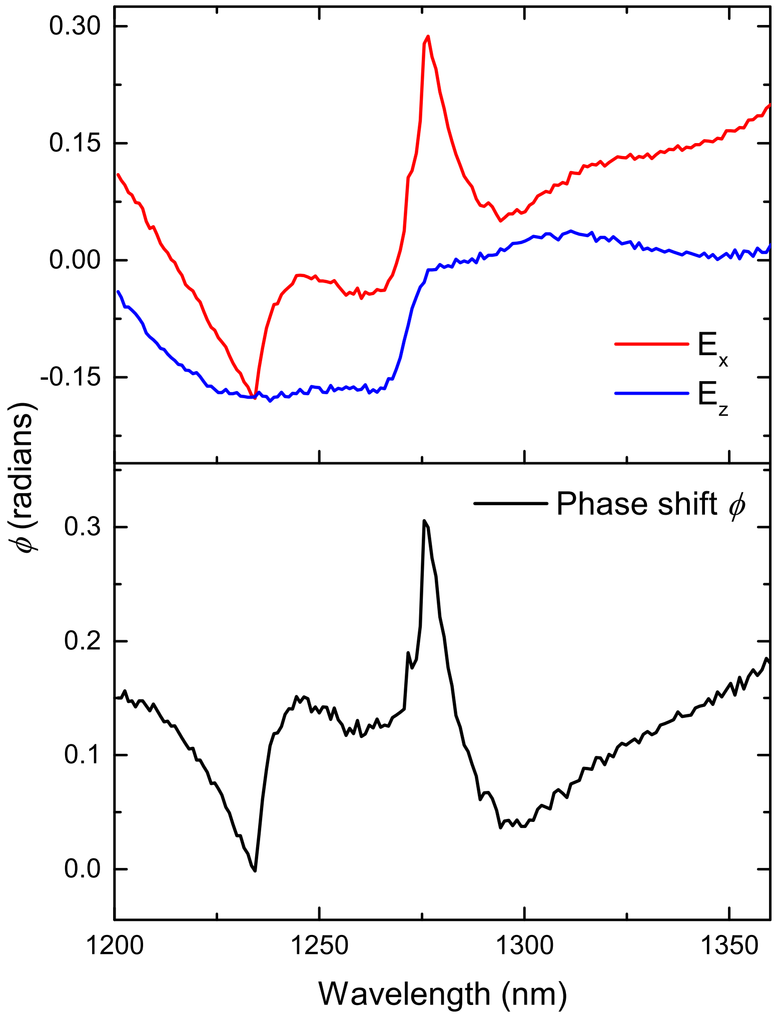

Figure S1: Numerically simulated spectral dependence of the phases (in the units of π) of the two projections of the electric field E_x, E_z at the Au/YIG:Co interface (top) and the phase shift φ between these projections (bottom). The narrow spike around 1275 nm corresponds to the SPP excitation at this interface.

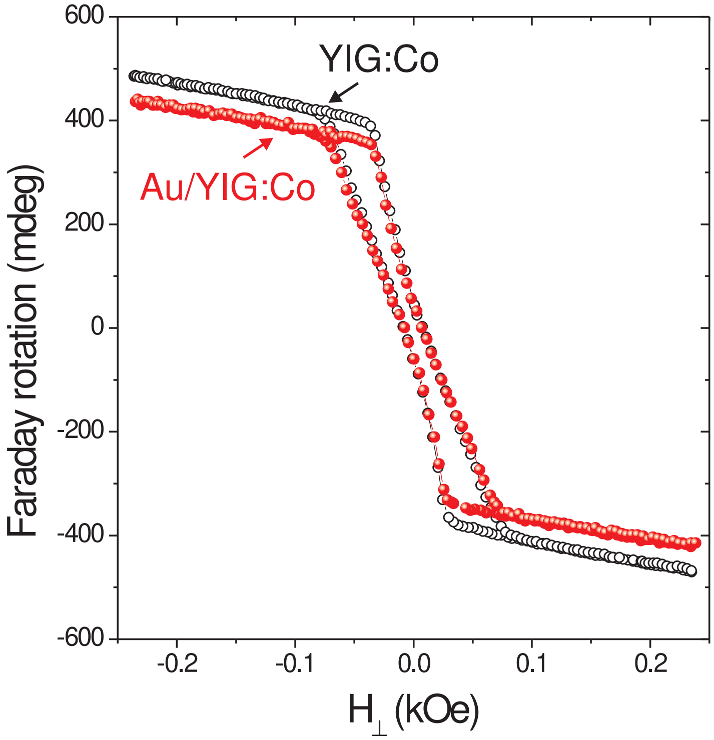

Figure S2: Experimental hysteresis loops of the Faraday rotation obtained on the bare YIG:Co garnet (black) and the Au/YIG:Co magneto-plasmonic grating (red) at the 800 nm wavelength. The external magnetic field is applied perpendicular to the sample plane. The slope is due to the paramagnetic contribution from the GGG substrate.
